# Knowledge, Skills, and Abilities for Managing Potentially Volatile Police–Public Interactions: A Narrative Review

**DOI:** 10.3389/fpsyg.2022.818009

**Published:** 2022-03-07

**Authors:** Craig Bennell, Bryce Jenkins, Brittany Blaskovits, Tori Semple, Ariane-Jade Khanizadeh, Andrew Steven Brown, Natalie Jennifer Jones

**Affiliations:** Department of Psychology, Carleton University, Ottawa, ON, Canada

**Keywords:** competencies, use-of-force, de-escalation, law enforcement, police training, public safety, non-escalation

## Abstract

We conducted a narrative review of existing literature to identify the knowledge, skills, and abilities (KSAs) necessary for officers who police in democratic societies to successfully manage potentially volatile police–public interactions. This review revealed 10 such KSAs that are frequently discussed in the literature. These KSAs include: (1) knowledge of policies and laws; (2) an understanding of mental health-related issues; (3) an ability to interact effectively with, and show respect for, individuals from diverse community groups; (4) awareness and management of stress effects; (5) communication skills; (6) decision-making and problem-solving skills; (7) perceptual skills; (8) motor skills related to use-of-force; (9) emotion and behavior regulation; and (10) an ability to treat people in a procedurally just manner. Following our review, we conducted semi-structured interviews (*N* = 7) with researchers who specialize in police training and adult education, interactions with individuals in crisis, and racialized policing, as well as two police trainers with expertise in de-escalation and use-of-force training. These interviews confirmed the importance of the 10 KSAs and highlighted two additional KSAs that are likely to be critical: understanding the role of policing in a free and democratic society and tactical knowledge and skills. To ensure that police–public interactions are managed effectively, police trainers may want to focus on the development and evaluation of these KSAs—something that is not always done currently.

## Introduction

Police officers are frequently involved in potentially volatile interactions with the public (Baldwin et al., 2016; Shjarback and White, 2016). A primary goal in such interactions should always be to minimize the potential for harm, which will often involve the use of non-escalation and de-escalation strategies by officers (Engel et al., 2020). When force is necessary to control these situations, the level of force used must be appropriate, given the totality of circumstances faced by the officer (Cyr, 2016), and the application of that force should never be based on extraneous factors that are unrelated to risk (e.g., citizen race).^1^

In addition to being able to apply a range of skills in a competent manner, police officers must consider a range of other issues when managing police–public interactions. These complexities are captured to some extent in models that officers are taught to draw on when making critical decisions. For example, in Canada, police officers are often trained to apply “NRA principles” during their interactions with the public (e.g., Peel Regional Police, 2015). This encourages officers to think about whether their decisions are *necessary* (e.g., to address the threat), *risk effective* (e.g., avoiding unnecessary harm to the individual, officer, and others), and *acceptable* (e.g., legally, publicly, and ethically). Thus, in jurisdictions where NRA principles are relied on, an optimal outcome in potentially volatile police–public interactions is one that is defensible across these multiple elements.

Training is provided to police officers in many countries to increase the likelihood that these sorts of outcomes can be achieved during challenging interactions with the public. For this training to be effective, not only must it adhere to principles of training and learning, but also it must arguably target relevant knowledge, skills, and abilities (KSAs).^2^ While some attempts have been made by researchers to articulate principles of training effectiveness and learning within the policing context (e.g., Angel et al., 2012; Andersen et al., 2017; Bennell et al., 2021; Jenkins et al., 2021), less effort has been invested in identifying exactly what KSAs to prioritize during police training.

The primary goals of this paper, therefore, are: (1) to begin the process of identifying the core KSAs that are relevant to the effective management of potentially volatile police–public interactions and (2) to determine the degree to which these KSAs have been empirically studied and supported. While we draw on international research to accomplish these goals, we are interested specifically in the implications of this review for policing in democratic societies, such as Australia, Canada, the United Kingdom (UK), and the United States (US). While we believe that the value of the identified KSAs, and the training that targets them, likely generalizes to other settings, the research and examples we draw on throughout this paper are heavily biased toward policing in these contexts.

## Methodology

To accomplish our goals of identifying relevant KSAs and determining the degree to which they have been studied and supported, we adopted a two-stage methodological approach, each of which will be described in this section.

### Narrative Literature Review

The first stage of our study was to conduct a narrative review of relevant literature using a two-step process. The first step of the narrative review involved an attempt to identify core KSAs highlighted in existing literature as being relevant to the management of potentially volatile police–public interactions in democratic policing contexts. In this first step, we conducted broad searches of 12 different databases (e.g., PsycINFO, Web of Science, and Scopus) using various keywords, as well as combinations of these keywords (e.g., knowledge/skills/abilities + non-escalation/de-escalation/use-of-force). The second step of the literature search involved a more targeted attempt to determine the degree to which the identified KSAs from the first step were supported by empirical evidence. In these more targeted searches, specific KSAs identified in the first step were used as key words (e.g., communication skills + de-escalation) so that empirical research related to these KSAs could be identified. For an article to be considered relevant to our review, it had to focus on a clearly defined KSA that may be relevant for managing police–public interactions, and it had to be examined within the context of public policing in a democratic society.

Given the lack of previous comprehensive reviews of such KSAs, a narrative literature review was deemed suitable. While less structured and comprehensive than a systematic literature review, narrative reviews not only allow researchers to gain an appreciation for the research landscape in an area, but they also lay the foundation for later systematic reviews. Narrative reviews, which are typically defined simply as a summary and critique of existing research, are common in the published literature, especially in fields like medicine (Baethge et al., 2019), and arguments have been made that they are valuable (Greenhalgh et al., 2018; Furley and Goldschmeid, 2021). However, there is also a danger that narrative reviews can be biased (e.g., cherry-picking studies to include in the review). To minimize this possibility, we paid particular attention to systematic reviews and meta-analyses when selecting literature to inform this paper.

### Subject Matter Expert Interviews

The second stage of our study involved semi-structured interviews with a small sample (*N* = 7) of subject matter experts (SMEs) following the completion of our review to determine: (1) whether they thought the KSAs we identified were important and (2) whether we missed important KSAs when conducting our review. The SMEs included researchers who had published extensively on training and education, police interactions with individuals in crisis, and racialized policing, as well as two police trainers with expertise in non-escalation, de-escalation, and the use of force. Interviews with these SMEs were completed either in person or *via* Skype by at least two interviewers, ranged from 90 to 180 min, and were audio-recorded so that we could refer to the conversation if required.

The basic structure for each interview was the same. Key terms were defined for the SME, such as non-escalation, de-escalation, and use-of-force, and then, each of the KSAs identified from the narrative literature review was presented one at a time. The SME was asked to speak to the relevance, or not, of each KSA for managing potentially volatile police–public interactions. The SMEs with expertise in responding to individuals in crisis and racialized community members were also specifically asked to reflect on the KSAs from that perspective. At the end of the interview, the SMEs were asked if they thought the list of KSAs was complete. If they did not, they were asked to provide additional KSAs that they felt were important. All SMEs agreed that the identified KSAs were important. However, the interviewees identified two additional KSAs that did not emerge from our review.

Each of the 10 KSAs that were identified in the narrative literature review are briefly described below and we highlight the state of empirical research surrounding each KSA, focusing as much as possible on previous systematic reviews and meta-analyses. Where systematic reviews and meta-analyses are not available, we draw from other types of research, including qualitative analyses, experimental studies, and randomized controlled trials. Following this, we discuss the two additional KSAs that emerged from the SME interviews. Beyond highlighting the complex nature of police–public interactions, and the competencies that are likely necessary to navigate the myriad challenges that might be encountered during these interactions, the KSAs identified in this study speak to issues that should arguably be targeted in training and help to clarify where current training gaps might exist. Accordingly, we conclude the paper by discussing the implications of our findings for police training and its evaluation.

## Officer KSAs Identified in the Literature

The broad KSAs that emerged from our literature review include: (1) knowledge of organizational policies and laws; (2) an understanding of mental health-related issues; (3) an ability to interact effectively with, and show respect for, individuals from diverse community groups; (4) awareness and management of stress effects; (5) communication skills; (6) decision-making and problem-solving skills; (7) perceptual skills; (8) motor skills, especially as they relate to the application of use-of-force; (9) emotion and behavior regulation; and (10) an ability to treat people in a procedurally just manner.

### Knowledge of Organizational Policies and Laws

Knowledge of relevant organizational policies and laws, and an understanding of how to apply this knowledge in operational settings, has been highlighted as critical for officers (e.g., Prenzler et al., 2013; Rajakaruna et al., 2017). It is beyond the scope of this paper to cover the full range of policies and laws that police professionals and academic researchers have highlighted as being important for effectively managing potentially volatile interactions between the police and the public. Instead, we provide several pertinent examples to clarify the content of this domain and to illustrate why this knowledge and understanding may be critical.

One example of why knowledge of organizational policies is important is that these policies typically dictate the courses of action available to officers working in a particular police service and the conditions under which certain response options are appropriate. For example, consider the use of conducted energy weapons (CEWs), which have been shown to be an effective use-of-force intervention option (e.g., Baldwin et al., 2017) and a useful de-escalation tool in some settings (e.g., Ho et al., 2011). Police services have different policies regarding the use of CEWs, such as whether using CEWs is permitted at all, who can use CEWs, under what conditions CEWs are allowed to be used, and so on. To ensure that the use of CEWs is congruent with expectations within any given police service, and to make sure they are used appropriately, researchers have suggested that officers need to be fully aware of, and understand, their organization’s CEW policies (e.g., Alpert and Dunham, 2010).

It has also been stressed that officers must understand the laws that dictate their policing authorities if they are to effectively manage potentially volatile situations. Laws governing the appropriate use of force are seen as being particularly important for officers to understand (e.g., Cyr, 2016). Contributors to this literature have also argued that officers should be familiar with significant pieces of legislation in the jurisdictions within which they operate, which fundamentally impact the ways in which the police can interact with people in crisis. Knowledge of legislation that regulates the administration of mental health care and specifies the criteria that need to be met for officers to take people experiencing mental health issues into custody is seen as being particularly important (e.g., Cotton and Coleman, 2008).

While we believe that knowledge of organizational policies and laws will have a significant, positive impact on an officer’s ability to interact effectively with the public, little research could be found that directly examines this KSA. For example, we could not locate any research that has compared the performance of officers who vary with respect to how much knowledge they possess around these matters. However, there is research indicating that organizational policy can positively influence police behavior, such as reducing their reliance on the use of force (e.g., White, 2001; Ariel et al., 2016; Jennings and Rubado, 2017; Shjarback et al., 2021). Organizational policies seem to have this effect by restricting the conditions under which certain actions (e.g., deadly force) can be taken by officers, thus limiting inappropriate officer discretion, and by providing police organizations or other bodies with the ability to formally address policy violations, which might act as a deterrent to other officers.

### An Understanding of Issues Related to Mental Health

Another KSA that has been increasingly highlighted in the literature is a deeper understanding of issues related to mental health (e.g., Cotton and Coleman, 2010; Jennings and Hudak, 2021). This KSA is particularly important given that interactions with persons who have a mental illness (PMIs) account for a significant percentage of police–public encounters in some jurisdictions (e.g., Brink et al., 2011; Boyce et al., 2015). Research suggests that officers do not always feel adequately prepared to handle potential crises involving PMIs (Reuland et al., 2009), and many police professionals and academic researchers have argued that police officers require additional training related to a broad range of mental health topics to address this issue. Suggested topics include the nature of mental illnesses, the tenuous relationship between mental illness and violence, the recognition of symptoms related to various mental illnesses, the sorts of strategies that can be utilized to effectively deal with behaviors exhibited by people experiencing mental health crises, and the likely reactions of PMIs to various police interventions (e.g., Lamb et al., 2002; Coleman and Cotton, 2014; Jennings and Hudak, 2021).

Relatedly, researchers have underscored the importance of officer attitudes with respect to mental health and PMIs. For example, it has been noted that an officer’s attitude toward PMIs (which will likely be shaped, in part, through training) is likely to influence their interactions with PMIs, including the outcome of these interactions (Watson et al., 2004; Watson and Angell, 2007). Much like they are in the broader community (e.g., Taylor and Dear, 1981; Corrigan et al., 2003), PMIs are frequently stigmatized by police officers. For example, one attitude that is relatively pervasive in some studies of police officers is that PMIs are likely to be dangerous (e.g., Penn et al., 1999; Ruiz and Miller, 2004; Watson et al., 2004). Even if an officer can accurately determine that they are interacting with a PMI, endorsing such a view will likely result in a suboptimal outcome to the interaction. Because perceived risk of threat plays a key role in an officer’s assessment of an encounter (see discussion of perceptual skills below), researchers have argued that reducing biases around mental illness may allow officers to interact with PMIs more effectively (Penn et al., 1999).

Another relevant KSA that has been highlighted relates to an officer’s knowledge and understanding of community mental health resources (Lamb et al., 2002). If police officers are unaware of community resources that can help support PMIs, or they do not know how to access them, situations that could potentially be resolved through appropriate referrals and diversions may escalate unnecessarily (e.g., Morabito, 2007; Morabito et al., 2012; Koziarski et al., 2021). Unnecessarily escalating an encounter can be costly, both in terms of officer and public safety, and in terms of police resources (e.g., more time will likely need to be dedicated to the case over time; Coleman and Cotton, 2010; Semple et al., 2021b).

Despite some mixed results, evaluations of mental health training for officers across North America and Europe where many of the above KSAs have been targeted, often report positive findings. For example, Seo et al. (2021) recently conducted a systematic review and meta-analysis of studies that examined the impact of Crisis Intervention Training (CIT) for police officers. Within these studies, there was a substantial effect on reported self-efficacy/confidence in responding to mental health calls (*M_d_* = 1.10, *k* = 5) and knowledge of mental illness (*M_d_* = 0.90, *k* = 8), and a moderate effect of CIT on attitudes toward PMIs (*M_d_* = 0.47, *k* = 8). They also found positive effects for time spent on scene with the individual as well as on the outcome of the call (*M_d_* = 0.94, *k* = 3 and *M_d_* = 0.40, *k* = 8, respectively). In contrast, the results for observed outcome measures, such as the use of force, arrests, and injury rates to officers and citizens, were less positive, ranging from −0.04 (injuries, *k* = 1) to 0.10 (use-of-force, *k* = 3). Despite the generally promising results reported in this meta-analysis, numerous methodological issues were raised about the studies being conducted on these topics; issues that warrant consideration when interpreting research that purportedly demonstrates the value of mental health training (Seo et al., 2021).

### Ability to Interact Effectively and Respectfully With Diverse Community Groups

Perhaps more than any other competency discussed in this paper, the KSA that seems to be occupying most of the public’s (and media’s) attention is an officer’s ability to interact effectively, and respectfully, with individuals from diverse community groups. If officers have biased views toward members of a particular group or are otherwise ill-equipped to interact appropriately with such members, undue conflict is likely to emerge (e.g., officers will be less likely to de-escalate such encounters effectively; Mastrofski et al., 1996; Tyler and Wakslak, 2004; Rosenbaum and Lawrence, 2017). While members of various communities have publicly addressed their tenuous relationship with the police (e.g., homeless young people persons who use substances, the LGBTQIA2+ community, PMIs, etc.; Baron, 2016; Israel et al., 2016; Krameddine and Silverstone, 2016), we focus here on interactions between the police and racialized communities given ongoing concerns about these relationships, which have been reignited since the death of George Floyd in May 2020 at the hands of a Minneapolis police officer (Hill et al., 2020).

Within North America, most of the available police research indicates that force is used disproportionately against members of racialized communities (e.g., Wortley, 2006; Fridell and Lim, 2016; Edwards et al., 2019; Wortley et al., 2020). This finding likely explains in part why members of racialized communities often express negative attitudes toward the police (e.g., Cheurprakobkit, 2000; Rosenbaum et al., 2005; MacDonald et al., 2007; Cao, 2011). As such, police professionals and researchers have argued that more time needs to be dedicated to developing KSAs that will improve police relations with racialized communities, and officer interactions with members of these communities (e.g., Shaffir and Satzewich, 2011; Schlosser et al., 2015; Spencer et al., 2016).

This will likely require the development of numerous KSAs. Included among those that have been emphasized in the literature is an understanding of historic and current police misconduct and how this may shape perceptions of the police among racialized groups, knowledge about how one’s place of origin may influence perceptions of the police, awareness of an officer’s own attitudes toward various racialized communities, an appreciation for cultural sensitivities, such as how body language may have alternative meanings across racialized groups, and an ability to reduce the expression of racial biases (e.g., Shusta et al., 2002; Ben-Porat, 2008; Schlosser et al., 2015; Spencer et al., 2016; Fridell, 2017).

Surprisingly, our search for literature examining such KSAs in the policing context identified relatively few studies. While research examining racial biases in various policing contexts was plentiful (e.g., traffic pullovers, stop and frisk, and police use of force; Lundman and Kaufman, 2003; Gelman et al., 2007; Paoline et al., 2018), well-controlled examinations of KSAs that might prevent such biases were much more rare. The research we could locate fell into two categories: assessments of cultural awareness (e.g., the importance of respecting diversity) and examinations of implicit bias (e.g., the importance of reducing the activation or managing the expression of implicit racial biases).

Only a small amount of research appears to be conducted on cultural awareness training for police officers, but studies that have been conducted support the value of this KSA. For example, Cornett-DeVito and McGlone (2000) found that two 4-h cultural awareness training sessions had a significant, positive effect on trainees with respect to attitudinal changes (e.g., being non-judgmental). This is generally consistent with evaluations from other fields. Importantly, these evaluations also suggest that such training can impact more than just attitudes; the training can also influence behavior at work. For example, in a meta-analysis of 65 studies, Kalinoski et al. (2013) found that diversity training in a variety of work contexts, including policing, positively influenced a range of affective-based (e.g., attitudes; *M_d_* = 0.27, 95% CI [0.21, 0.33], *k* = 44), cognitive-based (e.g., knowledge, *M_d_* = 0.62, 95% CI [0.52, 0.72], *k* = 25), and skill-based outcomes (e.g., on-the-job behavior; *M_d_* = 0.35, 95% CI [0.20, 0.50], *k* = 6).

The influence of implicit racial biases has been more extensively studied in policing, but only in a limited range of settings. The topic studied most relates to shoot/no shoot decision-making. In their meta-analysis of 42 such studies, Mekawi and Bresin (2015) found mixed results related to shooting biases depending on the way bias was operationalized. For example, when compared to White individuals, participants in these studies are significantly faster to shoot armed Black individuals (*M_d_* = −0.13, 95% CI [−0.19, −0.06], *k* = 32), slower to *not* shoot unarmed Black individuals (*M_d_* = 0.11, 95% CI [0.05, 0.18], *k* = 32), and have a more liberal shooting threshold for Black individuals (*M_d_* = −0.19, 95% CI [−0.37, −0.01], *k* = 29). In contrast, participants in these studies are not more likely, on average, to shoot more unarmed Black individuals as compared to White individuals (*M_d_* = −0.01, 95% CI [−0.11, 0.09], *k* = 28). Importantly, and of most relevance to the current review, when examining whether shoot/no shoot decisions are predicted by the prejudicial attitudes of the participants—measured as personal endorsements of stereotypes or knowledge of cultural stereotypes—the meta-analysis found “very small relations with shooter biases” (p. 128). This raises questions about whether the ability to manage prejudicial attitudes is an important KSA to possess within this context; however, the studies included in the meta-analysis lack ecological validity, so the extent to which any of these results generalize to field settings is unclear.^3^

That being said, evaluations of police training programs delivered in real-world settings that are designed to increase awareness of implicit biases also raise doubts about the role of implicit biases in police decision-making (or at least, the impact that implicit bias training can have on decision-making). In one recent study, Worden et al. (2020) carried out a randomized controlled trial of Fair and Impartial Policing (FIP) within the New York City Police Department. Their evaluation examined the effects of this training on officers’ beliefs and attitudes (compared to before the training) as well as its effects on enforcement actions. Their results were mixed, but generally not promising.

With respect to officers’ knowledge of implicit biases, moderate improvements were seen, although there was some decay observed in follow-up surveys conducted 2–13 months post-training. Only small improvements were observed in officers’ attitudes toward discrimination and their motivation to act without prejudice, potentially explained by the fact that, even prior to training, most officers already considered discrimination to be a significant problem and indicated they were motivated to act without bias. In terms of the real-world impact of the training, when officers were asked about the extent to which they applied the FIP training during the month prior to the survey, results revealed that 42% reported not using the training, 31% reported using it sometimes, and 27% reported using it frequently. Relatedly, when Worden and colleagues examined the impact of the training on reducing racial/ethnic disparities in enforcement actions, such as stops, frisks, searches, arrests, and applications of force, results did not reveal evidence that disparities were reduced for those that received the training.

### Awareness and Management of Stress Effects

The ability to recognize stress in oneself, understand how it will impact performance, and mitigate its effects have all been identified as important competencies that are apt to improve an officer’s ability to effectively manage potentially volatile police–public interactions (e.g., McCraty and Atkinson, 2012; Andersen et al., 2015; Andersen and Gustafsberg, 2016).^4^ Police frequently experience high levels of stress during these interactions (Anderson et al., 2002; Andersen et al., 2016; Baldwin et al., 2019) and a heightened stress response can have significant implications for how police officers respond. Indeed, based on our review of the literature, it is clear that while some level of stress can improve performance, significant amounts of stress can result in impairments to various aspects of police performance including shooting accuracy (e.g., Taverniers and De Boeck, 2014), quality of skill execution (e.g., Renden et al., 2014), proportionality of force applied (e.g., Nieuwenhuys et al., 2012), self-control (e.g., Haller et al., 2014), perceptual and attentional control (e.g., Giessing et al., 2019), memory (Hope et al., 2012), and communication (e.g., Arble et al., 2019).

Studies also generally support the fact that one’s awareness of stress effects, and more importantly, one’s ability to manage those effects, can improve an officer’s performance in potentially volatile police–public interactions (e.g., Oudejans, 2008; Nieuwenhuys and Oudejans, 2011; Arnetz et al., 2013). For example, training in environments which gradually increase the level of stress to develop stress resiliency in officers has been associated with improved performance during subsequent high-stress encounters (e.g., Oudejans and Pijpers, 2009; Nieuwenhuys and Oudejans, 2011) and programs that focus on relaxation and imagery training to develop an officers’ ability to modulate their stress response during potentially volatile police–public interactions can also improve performance (e.g., Arnetz et al., 2009; Andersen and Gustafsberg, 2016; Andersen et al., 2018). However, more research is certainly needed to ensure that performance improvements regularly transfer to field settings.

Meta-analytic studies also support the importance of these KSAs. For example, a meta-analysis of 37 studies conducted by Saunders et al. (1996), which examined the impact of stress-exposure training on performance in various domains (e.g., sport and medical), revealed that this training has a moderate impact on performance enhancement (*r* = 0.296, *p* < 0.001). A more recent meta-analysis that focused on pressure training, which is like stress-exposure training, reported similar results, including a moderate effect on performance for the three studies that were conducted in the law enforcement context (*g* = 0.63, 95% CI [−0.14, 1.39]; Low et al., 2020).

### Strong Communication Skills

The ability to communicate effectively is a KSA that has long been recognized by police professionals and academic researchers as a skill set that will likely assist police officers in preventing encounters from escalating unnecessarily and promoting effective de-escalation (e.g., McCamey and Carper, 1998; Kesic et al., 2013; Police Executive Research Forum, 2016). It has been proposed that both verbal and non-verbal communication can be used by those involved in potentially volatile situations to create clear expectations and accomplish negotiated positions of mutual benefit (Gertz, 1980; Lowe, 1992; Duperouzel, 2008). In the policing context, effective communication skills have been deemed especially important because they can be applied to gain voluntary compliance, hence decreasing the likelihood that officers will have to rely on force (Sun, 2003; Todak and James, 2018).

Much of what is known about the use of communication to effectively manage police–public interactions comes from fields outside of policing (e.g., the healthcare setting; Price and Baker, 2012; Richmond et al., 2012; Engel et al., 2020). However, researchers have argued that many of the same principles that apply to calming agitated individuals in other contexts (e.g., patients) are likely to apply to citizens with whom officers interact (e.g., Oliva et al., 2010; McDermott and Hulse, 2012). Researchers speaking to these issues highlight a series of communication strategies intended to facilitate calmness. Among other things, such strategies include: (1) showing empathy; (2) respecting individuals and their personal space to create safe and comfortable distances; (3) using appropriate body language, such as not concealing one’s hands or engaging in excessive staring, which may be interpreted as threatening; (4) establishing rapport (e.g., by introducing oneself) and ensuring that expectations are clear and that individuals are reassured that the intervener wants to help; (5) giving people options or choices to the extent possible; (6) being concise and using simple language to increase understanding and reducing the likelihood of the situation escalating due to confusion; (7) repeating messages, which can combat the effects of limited processing if the individual is agitated; (8) active listening, which involves the intervener demonstrating to the individual that they care and are truly listening, often through the use of verbal and non-verbal gestures (e.g., head nods, restating statements, paraphrasing, and summarizing); (9) setting limits (e.g., being clear about what behavior is and is not acceptable, and what the consequences for unacceptable behavior are); and (10) reducing stimulation (e.g., eliminating unnecessary distractions; Carlsson et al., 2000; Johnson and Hauser, 2001; Duperouzel, 2008; Price and Baker, 2012; Todak and James, 2018).

The limited research conducted within the policing context suggests that communication training can influence officer behaviors. For example, Krameddine et al. (2013) evaluated specially designed scenario-based training to improve police interactions with PMIs, much of which involved post-scenario feedback from instructors and the role players about the officers’ use of verbal and non-verbal communication strategies. While officer attitudes toward PMIs did not change as a result of training, there were tangible improvements seen in various real-world behavioral outcomes, including a significant increase in recognizing that mental health issues were the reason for a call, improved efficiency in dealing with mental health calls, and decreases in the use of weapons and physical force during interactions with PMIs. Similarly, during an evaluation of Chicago’s Quality Interaction Program, Rosenbaum and Lawrence (2017) found that officers who completed training that focused on communication skills, such as listening, empathy, and procedural justice, were more respectful and reassuring during role play scenarios and relied on force and arrests less often than those who did not receive the training.

Other, more recent evaluations of training programs that focus (at least to some extent) on improving officer communication have also revealed some positive results. For example, a randomized controlled trial of a social interaction training program conducted by McLean et al. (2020) found that trained officers placed high priority on procedurally fair communication during hypothetical police–public encounters. However, analyses did not show an effect of training on police use of force. This stands in contrast to the recent randomized controlled trial of Integrating Communications, Assessment, and Tactics training conducted by Engel et al. (2022). They report on many impacts of this training, including positive changes in officer attitudes, but also changes in actual officer behaviors, most notably significant reductions in the use of force (and associated reductions in citizen and officer injuries).

### Decision-Making and Problem-Solving Skills

Officers frequently rely on sound decision-making and problem-solving skills to perform successfully in the field (e.g., Kilner and Hall, 2005; Ward et al., 2011; Suss and Ward, 2012; Preddy et al., 2020). In many cases, decisions will have to be made in a split-second and require rapid problem-solving; at other times, more deliberate decision-making will be possible based on strategic problem-solving skills (Murgado, 2012). While important for most policing tasks, sound decision-making and problem-solving skills are especially important when deciding how to manage a potentially volatile police–public interaction.

The importance of sound decision-making and problem-solving in this context is made evident when one considers the concept of officer-induced jeopardy. Officer-induced jeopardy occurs when an officer fails to make tactically sound decisions, which ultimately places them (and potentially others) at an elevated risk of harm (Klinger, 2005; Cyr, 2016). In some cases, this can lead to a situation where the only viable option to resolve the incident is to use a high level of force (Adang, 2012); had the officer made a different series of decisions, less force (or no force) may have been sufficient for resolving the conflict (Klinger, 2005; Cyr, 2016). Examining data from a Belgian police force, Pauwels et al. (1994) identified several tactical “sins” committed by officers that increased the likelihood of a suboptimal outcome. Examples of such tactical sins include decisions to jump in front of approaching vehicles, not utilizing available cover, and giving chase at any price.

We were unable to locate any systematic reviews or meta-analyses on the topic of police decision-making and problem-solving; however, we identified a reasonably large volume of research pertaining to the application of these skills in the context of potential use-of-force encounters. One KSA that has received a considerable amount of attention is adaptive (or flexible) problem-solving. Collectively, research on this topic suggests that the problem-solving processes that experienced police officers engage in, both in simulated and real-world encounters, are often substantially different from the processes that characterize less experienced officers (e.g., Boulton and Cole, 2016; Heusler and Sutter, 2020; Mangels et al., 2020). Boulton and Cole (2016), for example, found that adaptability was a key factor that distinguished expert authorized firearms officers in the United Kingdom from more junior officers in that experienced officers demonstrated greater flexibility in responding to situational changes in complex incidents, whereas novice officers were bound by more rigid, sequential, and linear decision-making strategies, which tended to align with standard operating procedures. These results are consistent with other research that has revealed important differences in the decision-making and problem-solving abilities of experienced and novice police officers that lead to enhanced performance for experienced officers, such as the ability to anticipate outcomes of interactions (e.g., Tashman et al., 2006; Ward et al., 2011; Suss and Ward, 2013).

The training literature also appears to support the role of these competencies in achieving optimal outcomes in police–public encounters (Preddy et al., 2020). For example, McCombs (2015) examined use-of-force rates over time within the Columbus Police Service following the implementation of a problem-based learning approach for all police recruits. While controlling for unemployment and homicide rates within the city, as well as officer experience, McCombs found that for every hour of problem-based learning that officers received, use-of-force rates decreased by 17 incidents a year. These findings suggest that the training allowed officers to implement problem-solving more effectively during their interactions with the public, in turn reducing the need for officers to use force.

### Perceptual Skills

In any given interaction with a member of the public, a police officer is tasked with conducting an initial risk assessment of the situation, which is subject to continuous revision and updating as the situation unfolds (Canadian Association of Chiefs of Police, 2000). This risk assessment is crucial in guiding an officer’s response to the situation. Although the risk assessment process is based purely on the officer’s perception of the incident, the process is often guided by an overarching use-of-force model or framework (e.g., Canadian Association of Chiefs of Police, 2000). These frameworks represent various factors that an officer should consider when conducting a risk assessment to help ensure an appropriate and justifiable response to an incident. The capacity of an officer to conduct a comprehensive risk assessment affects their ability to appropriately formulate a response to prevent the escalation of an incident, to effectively de-escalate a situation, and/or to apply appropriate force when it is necessary to do so. Given that the entire risk assessment process is driven by an officer’s perception, it has been argued that advanced perceptual skills are an important KSA for officers (Dror, 2007; Tiesman et al., 2015).

To ensure that risk is accurately perceived, it has been argued that officers require high levels of situational awareness, which has been defined as “the ability to perceive and process all potential threats in the environment” (Andersen and Gustafsberg, 2016, p. 4). Situational awareness is thought to occur in three stages (Endsley, 1988). The first stage involves perceiving elements of the environment, the second stage involves understanding the meaning of the identified elements, and the final stage involves the projection of future events that may occur based on an understanding of the situation.

Despite the obvious importance of the risk assessment process for police decision-making during potentially volatile police–public interactions, this process has rarely been the subject of empirical scrutiny within the policing context. Consequently, we were unable to locate any systematic research on the topic of risk assessment. However, our literature search did uncover a small number of empirical studies that support the importance of perceptual skills generally, and situational awareness specifically. For example, with respect to perceptual skills, research has demonstrated that tactical officers are better able to discriminate between threatening (e.g., a gun) and benign (e.g., cellphone) stimuli during rapid shoot/no shoot scenarios compared to more novice officers (Vickers and Lewinski, 2012; Johnson et al., 2014). In part, this ability appears to be due to differences in where officers are focusing their attention; for example, tactical personnel spend more time looking at areas where weapons can be concealed or held (e.g., hands or waist; Heusler and Sutter, 2020) compared to novices who tend to focus on elements like the face of the individual with whom they are interacting (e.g., Vickers and Lewinski, 2012; Landman et al., 2016; Heusler and Sutter, 2020).

Several studies have also demonstrated positive effects of training programs, which seek to improve situational awareness during simulated encounters (e.g., Saus et al., 2006; Andersen and Gustafsberg, 2016; Andersen et al., 2018). Generally, this research suggests that officers who complete these programs have improved situational awareness (e.g., being able to identify more threat cues in the environment) compared to those in control groups, which results in better decision-making. For example, in one study, Saus et al. (2006) attempted to improve situational awareness during use-of-force events by providing 20 new Norwegian police recruits with this sort of training. Training involved participants taking part in scenarios delivered *via* a simulator. Within each scenario, a freeze technique was used whereby the video simulation was stopped during which time participants would be asked about the three phases of situational awareness (i.e., perception, comprehension, and projection); similar discussions were held during debriefings. Those recruits who received training were compared to a control group of 20 new recruits who received more traditional marksmanship training on the simulator. Among other findings, trained recruits demonstrated improved situational awareness and improved decision-making (e.g., shot frequency and hit rate) in a shoot scenario compared to recruits in the control group. Similar results have been reported more recently by Andersen and Gustafsberg (2016).

### Motor Skills Related to Use-of-Force

While the use of force should be avoided whenever possible, police officers clearly need to develop the ability to effectively apply force when required to do so. Indeed, the ability to efficiently and effectively gain control of an individual may be crucial when attempts to de-escalate have failed or are not feasible. Under these circumstances, an officer must sometimes use force to protect themselves or others. Appropriate levels of force can also prevent a situation from escalating further, thus reducing the overall potential for harm. For example, as mentioned previously, Ho et al. (2011) examined the use of CEWs within a hospital setting. Ho et al. (2011) found that simply using the laser sighting device, which projects red dots on the individual, prevented interactions with agitated patients from escalating. Ideally, officers should also know what intervention options work best in any given set of circumstances, with respect to both effectiveness and safety (Baldwin et al., 2018; Baliatsas et al., 2021; Semple et al., 2021a).

While recent real-world tragedies, including the recent death of Daunte Wright (Nickeas, 2021) in the United States,^5^ clearly highlight the need for officers to have mastery of their use-of-force intervention options, we were unable to locate any systematic reviews, meta-analyses, or experimental research that specifically examined these sorts of KSAs. However, some research supports the view that proficiency in the use of intervention options will likely be associated with numerous benefits, including enhanced public and officer safety. For example, it has been found that injuries to both citizens and officers are more likely when the officer needs to apply additional force after an initial intervention was insufficient at gaining control of the individual (e.g., Adedipe et al., 2012). Similarly, the longer it takes for an officer to gain control of an individual, the greater the likelihood of both parties becoming injured (e.g., Castillo et al., 2012). Other research has found that the longer a conflict between an officer and an individual lasts, the more likely an officer is to use force, even when the individual is displaying non-violent resistance (Alpert et al., 2004; Wolf et al., 2009). Thus, based on existing research, it seems relatively clear that when officers can quickly gain control of resistant or aggressive individuals through the appropriate and effective use of force, officers are likely to prevent the encounters from escalating further, ultimately requiring less force, which likely results in fewer injuries.

It may also be worth highlighting that if officers are able to gain control of a resistant or aggressive individual efficiently and effectively without using force, or using lower levels of force, not only are injury rates to both parties likely to be reduced, but the optics of the encounter will likely improve. Research suggests that the perceived legitimacy of police use-of-force is related to the public’s approval of the police (Tuch and Weitzer, 1997; Kaminski and Jefferis, 1998; Weitzer, 2002). Thus, if the public views an officer’s use of force as excessive (e.g., because the officer uses force multiple times or escalates their level of force because the initial application of force was ineffective) or biased (e.g., because the officer’s use of force seems to be influenced by irrelevant factors, such as the citizen’s race), perceptions of the police are likely to be compromised. Therefore, it is arguably more beneficial, from both a safety perspective *and* a public relations perspective, for an officer to use an effective intervention option once than to use additional force incrementally. In cases where multiple applications of force are used, any recording of the event that surfaces, and the media attention that inevitably follows, will likely only allow viewers to focus on the quantitative aspects of the encounter (e.g., the number of force applications) rather than qualitative components (e.g., the initial application of force was ineffective, but the perceived threat persisted, thus necessitating additional force). As such, efficient and effective force by officers may also improve the perception of any use-of-force encounter.

### Emotion and Behavior Regulation

Police are routinely exposed to situations that elicit intense emotions, but which require a controlled response (Berking et al., 2010). By its nature, police work can be dangerous and unpredictable, and it will certainly involve many interactions with individuals who can be anxious, angry, upset, or confused. Officers are tasked with regulating their own emotions and behaviors to make sound decisions during their interactions with the public, regardless of how antagonistic the situation becomes. In many cases, this may allow officers to accomplish important goals (e.g., de-escalating an encounter; Baumeister et al., 2006, 2007). Indeed, Tangney et al. (2004) found that those with higher self-control are better able to inhibit undesirable responses and accomplish their goals in high pressure situations relative to those with lower self-control. Therefore, emotion regulation and behavioral control are likely implicated in an officer’s ability to de-escalate and make appropriate use-of-force decisions in the field.

Consistent with this, the need to remain “above” the conflict during tense situations has been highlighted by police as an important KSA (Murphy, 2009; Rajakaruna et al., 2017) and this is supported by the de-escalation literature. For example, in their review of the literature examining key components of de-escalation in healthcare settings, Price and Baker (2012) found that maintaining personal control was particularly important. Furthermore, self-control has been associated with enhanced decision-making in both simulated and real-world settings within policing (e.g., Brown and Daus, 2015; Donner et al., 2017) and non-policing contexts (de Ridder et al., 2012). For example, in one study, police who completed a task that was previously shown to reduce one’s self-control (i.e., the cold pressor task; Hagger et al., 2010) were faster to initiate aggressive action toward a role player (Staller et al., 2018). Some researchers have also created proxies for self-control using background information on police officers (e.g., whether the officer had ever had their driver’s license suspended) to examine the relationship between complaints against officers and the use of force (Donner and Jennings, 2014; Donner et al., 2017). These studies suggest that lower ratings of self-control are positively associated with officers receiving public complaints for verbal and physical abuse, as well as being involved in police shootings.

One way to increase self-control has been referred to as “thinking slow” (i.e., engaging a cognitive system described as conscious, controlled, deliberate, effortful, statistical, and suspicious; Kahneman, 2011). In contrast, “thinking fast” is automatic, intuitive, unconscious, and effortless, and is largely governed by emotions (Brown and Daus, 2015). The fast-thinking system, while obviously important, particularly for potentially risky split-second situations that require quick, adaptive responses, is prone to systematic errors (Schleifer, 2012). This is likely because the fast-thinking system appears to be largely controlled by one’s schemas and stereotypes, which can result in serious mistakes if one is not careful (e.g., unintentionally acting on a racial bias).

Given the potential consequences of “thinking fast,” Owens et al. (2018) evaluated a program that sought to help officers develop their ability to “slow down” their thinking during interactions with the public. After being involved in an incident in which a report had been filed or an arrest had been made, 221 officers were randomly assigned to participate in a supervisory meeting while 1,213 officers acted as the control group and did not partake in a meeting. During the meeting, officers were asked a series of open-ended questions designed to get them to slow down and reflect on the decisions they had made on scene (e.g., did they incorporate new information into their understanding of what occurred as the event unfolded, or did they act on autopilot?). Six weeks after the supervisory meeting, officers were 12% less likely to make an arrest and between 16 and 50% less likely to be involved in a use-of-force event compared to those in the control condition. Owens and colleagues concluded that a minor supervisory intervention that encourages officer to slow down their thinking may result in substantive changes in how police and citizens interact with each other. They note that there will always be situations where automated responses (i.e., fast thinking) and punitive outcomes are necessary. However, they argue that, “…to the extent that (otherwise efficient) automation can occasionally lead to the escalation of an encounter, officers who continue to gather and process all information available on scene [i.e., engage in slow thinking] … may be able to recognize and diffuse tense situations sooner” (pp. 48–49).

### Treating Citizens in a Procedurally Just Manner

Police professionals and academic researchers, particularly in Australia, the United Kingdom, and the United State, have dedicated much effort over the last several decades to understanding how an officer’s treatment of a citizen during their interaction can impact the encounter (McCluskey et al., 1999). More specifically, research has examined how treating citizens in a procedurally just manner influences outcomes of encounters. Given mounting evidence demonstrating that the degree of procedural justice shown by officers during encounters with the public positively influences citizen behavior, it has been argued that this is likely to be a KSA that could enhance an officer’s ability to effectively manage potentially volatile police–public interactions (e.g., Mastrofski et al., 1996; Watson and Angell, 2007; Dai et al., 2011).^6^

Procedural justice is generally thought to include several key components. According to Mazerolle et al. (2013), procedural justice in the context of police–public encounters focuses on: (1) dialogue that encourages citizen participation during the interaction prior to an officer reaching a decision (what is called “citizen voice”); (2) neutrality in an officer’s decision-making; (3) the expression of dignity and respect throughout the encounter; and (4) attempts to convey to citizens that the officer has trustworthy motives. Research indicates that officers who interact with citizens in a procedurally just manner can generate positive outcomes, such as citizens being more likely to comply with officer requests (e.g., Dai et al., 2011; Walters and Bolger, 2019; Wolfe et al., 2019). Relatedly, other research has shown how procedural justice principles may improve an officer’s ability to de-escalate situations or prevent them from escalating in the first place (e.g., Rosenbaum and Lawrence, 2017).

Evaluations of procedural justice training have also generally produced positive results, suggesting that an officer’s ability to draw on principles of procedural justice during their interactions with the public is an important KSA. For example, in a systematic literature review of 28 studies, Mazerolle et al. (2013) found that, regardless of the initiative, the public reported an increased willingness to cooperate and comply with police requests in areas where legitimacy-enhancing programs were implemented, in addition to voicing a greater degree of general satisfaction with the police.

## Officer Competencies Identified by Subject Matter Experts

During the interviews, all the SMEs agreed that officers should possess the KSAs identified in our narrative literature review if they are to be able to effectively manage potentially volatile police–public interactions. However, as previously mentioned, they identified two additional sets of KSAs that did not emerge from our review, which they deemed critical: understanding the role of policing in a democratic society and tactical knowledge and skills.

### Role of Policing in a Democratic Society

Issues related to the role of policing in a free and democratic society were discussed by several SMEs, revealing a political dimension to managing potentially volatile police–public interactions, and a very compelling case was made that this should be seen as a core KSA for all police officers. The SMEs not only spoke to the importance of developing a practical understanding of the laws governing policing, but also expanded upon this to include a deeper appreciation for issues that extend beyond typical laws that might be emphasized in non-escalation, de-escalation, and use-of-force training, such as local, federal, and international declarations of human rights.

The SMEs also noted the connections between knowledge of these issues and the other KSAs discussed above, such as the competencies related to the policing of diverse communities, including PMIs and racialized citizens. While a deeper understanding of the role of policing in a democratic society cannot eliminate the stigma or biases that some police officers might hold, it does highlight the importance of minimizing stigma and biases as much as possible; moreover, it encourages officers to interact with all members of the public in a fair, respectful, and compassionate fashion, and to always prioritize the sanctity of life.

Although we could not locate any empirical research related to this KSA, it is easy to generate common-sense arguments for why police training should focus on these issues. Developing this KSA through training could logically have a positive impact on how officers manage potentially volatile police–public interactions.

### Tactical Knowledge and Skills

Another KSA identified by many of the SMEs that was deemed critical pertains to knowledge of various tactics—such as the effective use of time, distance, cover, and concealment—and the ability of officers to use these tactics effectively during stressful, dynamic interactions with the public. While most of the discussion surrounding sound tactics was found in professional magazines and other outlets (e.g., websites, such as *policeone.com*), which explains why this KSA did not emerge from our literature review, we were able to locate some empirical research on this topic during focused searches conducted following the SME interviews.

Most of the research we found focused specifically on how sound tactics can enhance officer safety in already escalated situations; little systematic research seems to exist to show how tactical knowledge and skills allow police officers to prevent interactions with the public from escalating in the first place. For example, in one study, Sandel et al. (2021) highlighted how moving off the line of attack (i.e., stepping to the side) significantly decreased the number of times a knife wielding individual could make contact with an officer before the officer fired their weapon. In another example, Blair et al. (2019) examined room clearing techniques and found that officers were less likely to be incapacitated from being shot when the officer rapidly entered the room as opposed to leaning out from behind cover or concealment.

## Implications for Police Training and Evaluation

The current paper represents a first step in identifying a broad set of KSAs that may be relevant to the management of potentially volatile police–public interactions. While many of the KSAs we identified were supported by a significant amount of empirical research (e.g., the importance of understanding mental health issues), the research surrounding other KSAs is currently underdeveloped (e.g., the importance of managing implicit biases). Conducting additional research on such KSAs should be an immediate priority and carrying out systematic reviews and meta-analyses on those KSAs where adequate research does exist must also be a matter of urgency. Until this work is done, we must be cautious about the weight we put on some of the KSAs highlighted in this paper even though the unanimous endorsement of each KSA by the SMEs interviewed in the current study (as well as by the policing scholars cited throughout this article) suggests these KSAs are likely to be important for effectively managing police encounters with the public.

This last point raises interesting questions about the value of the current project. Is it a surprise that the SMEs unanimously endorsed the KSAs described above? Would anyone doubt that the police need these KSAs to manage police–public interactions? We do not think it is particularly surprising that the SMEs agreed that the identified KSAs are important now that they have been generated through the literature review, nor do we have reason to believe that others would doubt the value of these KSAs. So, given this, what does this review and the SME interviews contribute to existing literature?

As we highlighted above, the reason we think this exercise was important is that it highlights the complex nature of police–public interactions and the very broad set of KSAs that are likely needed to manage these encounters effectively and ethically. The review also allows us to begin speaking to the sorts of KSAs that should arguably be targeted in training and evaluation, and to clarify where current training and evaluation gaps exist, endeavors that would be challenging without first identifying the relevant KSAs. We need to identify applicants and officers who do not possess (or do not sufficiently possess) all relevant KSAs to appropriately train them on the competencies they lack.

A key priority with respect to training will be to determine the extent to which programming currently in place within police services maps onto the sorts of KSAs highlighted in this paper. Engaging in this process will provide agencies with valuable insights into ways they can modify their training to enhance its effectiveness. For example, our observations of training over the last several years within North American police services suggests that the sorts of tactical skills highlighted above are frequently focused on in training. However, other competencies discussed above are not focused on to the same extent, or at all, even though they are likely to be critical. For instance, we do not know of many North American police services who provide adequate training on stress awareness and management. This is particularly alarming given that other important skills, such as the ability to effectively communicate with potentially violent citizens or to regulate emotions and behaviors, will be more challenging if the officer involved cannot effectively manage their stress. The lack of training in this area is also concerning because good training options arguably do exist (e.g., the iPrep program for modulating physiological stress; Andersen et al., 2018).^7^

Once progress on this mapping exercise has been made, police services will also need to determine how to best tackle the range of KSAs that are highlighted above, assuming future research confirms their importance. In addressing this challenge, several key questions will need to be answered. For example, when should the training for a given KSA take place (e.g., during basic training, in-service training, or both)? How frequently should the training occur to minimize KSA perishability (e.g., once, every training cycle, every other training cycle)? And what methods should be used to deliver the training (e.g., classroom instruction, learning from people with lived experience, scenario-based training, a combination of all these methods)?

Several attempts have been made to articulate effective methods for training delivery in the policing context, which may help answer some of these questions, including methods for training officers on how to manage potentially volatile police–public interactions (e.g., developing scenario-based training for skills acquisition; Bennell et al., 2021; Jenkins et al., 2021). In addition, as mentioned above, some potentially useful training programs already exist for some of the KSAs that emerged from our review, including stress management (e.g., Andersen et al., 2018), interpersonal communication (e.g., Engel et al., 2022), and an understanding of mental health-related issues (e.g., Krameddine et al., 2013). However, there are still significant gaps in the research, which make it challenging to answer the types of questions presented in the previous paragraph, especially for certain KSAs (e.g., emotion and behavior regulation, managing racial biases, and adaptive problem-solving). Therefore, police researchers and police practitioners need to work together to undertake research targeting these questions.

This review also has implications for a related activity that needs urgent attention—the evaluation of non-escalation, de-escalation, and use-of-force training. Police services, generally speaking, do not adequately attend to this task (Bradley and Connors, 2007). This is problematic, even in cases where the training being offered has been validated in other jurisdictions. As we have argued elsewhere (Bennell et al., 2021), there are many reasons why a training validation study may not generalize across jurisdictions, including but not limited to differences in trainee skills, training resources, and trainer qualifications. Evaluations of training should involve police services systematically monitoring their own training to determine whether important KSAs of the sort described above are being developed and modifying said training as needed to ensure it remains effective.

What form should this evaluation take? The process ought to be informed by the outcomes that the training is specifically designed to impact (Bradley and Connors, 2007). In addition, the process of evaluating the KSAs should be made as objective as possible. This can be done by relying on carefully crafted “assessment models,” which include detailed scoring rubrics (e.g., Norris and Wollert, 2011; Vila et al., 2018). Finally, given that the goals of non-escalation, de-escalation, and use-of-force training are multifaceted, and may include elements like knowledge acquisition, attitude and behavior change, and even organizational or community impact, evaluation tools will be required to effectively evaluate these various goals. Such assessments will prevent the need to make inferences about higher-order outcomes (e.g., whether behavior has changed) from evaluations of lower-order outcomes (e.g., whether knowledge has been acquired), which is critical given evidence that lower-order outcomes do not predict higher-order outcomes well (Alliger et al., 1997; Arthur et al., 2003; Saks and Burke, 2012). Moreover, different types of KSAs appear to degrade at different rates (e.g., knowledge degrades faster than skills; Arthur et al., 1998), so it is important to assess these outcomes separately.

## Conclusion

The current narrative literature review and SME interviews provide preliminary support for numerous KSAs that officers should possess to manage potentially volatile police–public interactions more effectively. We believe that this list of KSAs can provide guidance to police agencies with respect to training and evaluation targets. That being said, it is currently still unclear for some of the KSAs what the exact impact will be on police–public interactions. Elucidating these points should be a priority for police researchers working in this area and for police services. Once this is done, evidence-based training can be developed, and training evaluations carried out, to ensure that police officers possess the necessary KSAs to interact with members of the public in a safe, ethical, and effective manner across the full spectrum of situations in which these interactions take place.

## Author Contributions

CB conceptualized the project, secured the funding, oversaw the review, and wrote the first draft of the article. BJ conceptualized the project, secured the funding, assisted with the review, and edited the article. BB conceptualized the project, secured the funding, assisted with the review, and edited the article. TS assisted with the review and edited the article. A-JK assisted with the review and edited the article. AB assisted with the review and edited the article. NJ assisted with the review and edited the article. All authors contributed to the article and approved the submitted version.

## Funding

This research was supported by a contract from Ontario’s Ministry of Community Safety and Correctional Services, which was awarded to CB, BJ, and BB, and a grant from the Social Sciences and Humanities Research Council (#435-2017-1354), which was awarded to CB.

## Conflict of Interest

The authors declare that the research was conducted in the absence of any commercial or financial relationships that could be construed as a potential conflict of interest.

## Publisher’s Note

All claims expressed in this article are solely those of the authors and do not necessarily represent those of their affiliated organizations, or those of the publisher, the editors and the reviewers. Any product that may be evaluated in this article, or claim that may be made by its manufacturer, is not guaranteed or endorsed by the publisher.

## Footnotes

^1^There is a lot of disagreement on how to define some of the terms highlighted in this paragraph (e.g., Todak, 2017). However, for the purpose of this report, *non-escalation* will refer to attempts by a police officer to prevent the escalation of conflict, tension, or harm in a police–citizen encounter. *De-escalation* will refer to attempts by a police officer to reduce conflict, tension, or harm in an encounter that has already escalated. Finally, *use-of-force* will refer to the use of any intervention by a police officer to compel compliance in an individual.

^2^By *knowledge*, we are referring to a theoretical or practical understanding of an issue, whereas we treat *skills* as specific proficiencies that are developed through experience or training and *abilities* as innate qualities that allow officers to successfully complete a relevant task.

^3^It is important to point out that several studies conducted since the time of this meta-analysis are more ecologically valid (e.g., using interactive simulations of police–citizen encounters rather than static images of individuals presented on a desktop computer). Particularly noteworthy are the studies carried out by James and her colleagues. Interestingly, they report results that are drastically different from many of the studies included in the reported meta-analysis (e.g., evidence for a shooting bias against White individuals rather than Black individuals; James et al., 2016). Consistent with this meta-analysis, however, James et al. (2016) found that measures of implicit biases were not predictive of shooting biases.

^4^Although not examined to the same extent, the ability to recognize stress in others may also be an important KSA. This would likely fall in another domain, likely the perceptual skills discussed below, given that an assessment of the individual, which is largely based on the perceptions of the officer, is commonly included in many of the risk assessment models that police officers rely on. See that section for a discussion of risk assessment.

^5^In the case of Daunte Wright, Officer Kimberly Potter fatally shot the victim when she intended to reach for her CEW.

^6^It should be noted that there are long standing debates about whether the value of procedural justice principles is culturally specific (e.g., Sun et al., 2017).

^7^We suspect that one of the reasons why these sorts of training programs are not being implemented is because the training is often lengthy. This is a challenge for most police services in North America given that the amount of time dedicated to training after an officer undergoes their initial cadet/recruit training is very limited. For example, once on active duty, it has been estimated that officers in the US receive on average less than one week (i.e., 35 h) of training per year (Reaves, 2010). While we could not locate similar data for Canada, we believe that the situation here is very similar.
